# The effect of ambient temperature, habitat quality and individual age on incubation behaviour and incubation feeding in a socially monogamous songbird

**DOI:** 10.1007/s00265-016-2167-2

**Published:** 2016-07-05

**Authors:** Seyed Mehdi Amininasab, Sjouke A. Kingma, Martje Birker, Hanno Hildenbrandt, Jan Komdeur

**Affiliations:** 1 Behavioural and Physiological Ecology, Groningen Institute for Evolutionary Life Sciences, University of Groningen, P.O. Boxs 11103, 9700 CC Groningen, The Netherlands; 2 Department of Environmental Science, Behbahan Khatam Alanbia University of Technology, Behbahan, Iran

**Keywords:** Age, Ambient temperature, Blue tit *Cyanistes caeruleus*, Habitat quality, Incubation, Incubation feeding

## Abstract

**Abstract:**

Incubation is an important aspect of avian life history. The behaviour is energetically costly, and investment in incubation strategies within species, like female nest attentiveness and the feeding by the non-incubating partner during incubation, can therefore vary depending on environmental and individual characteristics. However, little is known about the combined effect of these characteristics. We investigated the importance of ambient temperature, habitat quality, and bird age on female incubation behaviour and male feeding of the incubating female (incubation feeding) in blue tits *Cyanistes caeruleus*, a socially monogamous songbird. An increase in ambient temperature resulted in a higher nest temperature, and this enabled females to increase the time off the nest for self-maintenance activities. Probably as a consequence of this, an increase in ambient temperature was associated with fewer incubation feedings by the male. Moreover, in areas with more food available (more deciduous trees), females had shorter incubation recesses and males fed females less often. Additionally, males fed young females more, presumably to increase such females’ investment in their eggs, which were colder on average (despite the length of recesses and female nest attentiveness being independent of female age). Male age did not affect incubation feeding rate. In conclusion, the patterns of incubation behaviour were related to both environmental and individual characteristics, and male incubation feeding was adjusted to females’ need for food according these characteristics, which can facilitate new insights to the study of avian incubation energetics.

**Significance statement:**

Parents often invest a substantial amount of energy in raising offspring. How much they do so depends on several environmental factors and on the extent they cooperate to raise the offspring. In birds, males can feed incubating females, which may allow females to stay longer on the nest, which, in turn, may ultimately improve reproductive success. The interplay between environmental factors and such incubation feeding on incubation attendance has, however, received little attention. Here, we show that favourable circumstances (higher ambient temperature and food availability) allowed incubating blue tit females to increase the time off the nest to improve self-maintenance and males to feed them less, whereas males also fed inexperienced partners more often. Thus, we show a concerted effect of several environmental and intrinsic factors on parental effort during incubation, which will help to improve the general understanding of avian incubation and parental care.

## Introduction

Understanding why species differ in patterns of parental care is important in evolutionary ecology studies (Clutton-Brock 1991). Incubation behaviour is an important aspect of avian parental care. Incubation is costly, and the magnitude of such costs can vary per species and depend on environmental and individual characteristics (Schultz 1991; Tinbergen and Williams 2002; Reid et al. 2002, 2003).

Incubating females usually face a trade-off between spending time on the nest to incubate the eggs to promote embryo development and time off the nest for investment in self-maintenance (Ardia et al. 2009). As such, incubating females are often constrained in their nest attentiveness because of limited access to energy resources while incubating (White and Kinney 1974; Martin 1987), but females may adjust to this limitation by optimizing their incubation rhythm (Conway and Martin 2000). The incubation rhythm consists of alternated periods, where females alternate leaving the clutch to obtain food (off-bouts) and returning to re-warm the eggs (on-bouts; Boulton et al. 2010). The incubation rhythm may therefore not only affect females’ condition but also the egg temperature and, hence, the embryo development (Lyon and Montgomerie 1987; Williams 1991).

In many species, particularly those with female-only incubation, males feed the incubating female, which may enable the female to increase nest attentiveness, and this may in turn affect the pair’s reproductive success (Martin and Ghalambor 1999; Hatchwell et al. 1999; Tewksbury et al. 2002; Olson et al. 2006). As such, the most important hypothesis for incubation feeding behaviour by males is the ‘female nutrition’ hypothesis (von Haartman 1958; Royama 1966), which states that incubation feeding enables the female to increase the time spent incubating (Matysioková and Remeš 2010; Matysioková et al. 2011; Ibáñez-Álamo and Soler 2012). If so, male incubation feeding can be seen as an indirect form of parental care (Klatt et al. 2008; Matysioková et al. 2011). Hence, the study of male incubation feeding and understanding its variation within and between species is also important when studying incubation behaviour (Lyon and Montgomerie 1987; Martin and Ghalambor 1999).

Although incubation may be affected by incubation feeding, both variation in incubation behaviour and incubation feeding may also be affected by environmental characteristics. The most important factors shown to play a role in explaining variation in incubation and male incubation feeding are environmental factors such as ambient temperature and food availability related to habitat quality (Zanette et al. 2000; Eikenaar et al. 2003; Pearse et al. 2004; Arnold 2011). Ambient temperature influences energy expenditure during incubation (Haftorn and Reinertsen 1985; Reid et al. 1999; Tinbergen and Williams 2002; Cresswell et al. 2004) because with lower ambient temperatures incubation time must be increased to keep eggs within temperature ranges for successful embryo development (Webb 1987; Sanz 1997; Matysioková and Remeš 2010). The intensity of male incubation feeding may therefore increase (but can at the same time be predicted to decrease) if decreasing ambient temperature leads to reduced food availability (Zanette et al. 2000). In addition to ambient temperature, habitat quality (also reflecting food availability) may have an effect on female nest attentiveness (Rauter and Reyer 1997; Zimmerling and Ankney 2005), recess length (off-bouts, Cucco and Malacarne 1997; Eikenaar et al. 2003; Duncan-Rastogi et al. 2006; Chalfoun and Martin 2007) and male incubation feeding (Zanette et al. 2000; Matysioková and Remeš 2010). Females in high-quality territories with higher food availability can spend more time incubating, potentially because their foraging efficiency is higher (Rauter and Reyer 1997; Conway and Martin 2000; Zanette et al. 2000; Zimmerling and Ankney 2005). Thus, several studies have shown that environmental characteristics can be strong predictors of incubation and incubation feeding behaviour.

In addition to environmental characteristics, individual characteristics like age and quality can have an influence on female incubation behaviour (Soler et al. 2001; Hanssen et al. 2002; Badyaev et al. 2003; Ardia et al. 2006) and male incubation feeding (Lifjeld et al. 1987; Siefferman and Hill 2005). Older or higher quality females may spend more time incubating the eggs (Ardia and Clotfelter 2007). Similarly, older males may be more capable of increasing the frequency of feeding the female during incubation (Lifjeld et al. 1987; Siefferman and Hill 2005). One potential explanation for age affecting incubation and incubation feeding may be that older individuals have a higher foraging efficiency (Pugesek and Diem 1983; Sæther 1990; Wunderle 1991).

As the above-mentioned examples illustrate, incubation behaviour and incubation feeding can be affected by several components, but studies that simultaneously investigate the combined effects of ambient temperature, habitat quality and individual age on both incubation behaviour and incubation feeding are scarce in songbirds (but see Boulton et al. 2010; Matysioková and Remeš 2010 for exceptions). In this research, we investigate the simultaneous effect of these factors on different measures of female incubation behaviour (nest attentiveness, recess length and nest temperature) and male incubation feeding in blue tits *Cyanistes caeruleus*, a socially monogamous songbird with female-only incubation and male incubation feeding behaviour. We predict a longer period of female nest attentiveness, a shorter recess length, a higher minimum nest temperature and a higher intensity of male incubation feeding when the ambient temperature is lower, when individuals are older and when individuals occupy higher quality habitats.

## Material and methods

### Study area and study population

The study was conducted during the breeding season of 2014 (March–June) in a nest-box breeding blue tit population at ‘De Vosbergen’ estate near Groningen in the north of the Netherlands (53° N, 06° E). The 54-ha study area consists of mixed deciduous and coniferous forest interspersed with areas of open grassland and contains 209 nest-boxes (with a 26 mm entrance hole; ca. 50 m apart) designed especially for blue tits.

### Breeding data collection

Blue tits were studied following the protocols of the long-term monitoring programme established for the study population since 2001 (for details see Korsten 2006). From the end of March, all nest-boxes were checked at least once a week to determine laying date and clutch size. Laying date was estimated by counting back from the observed clutch size, assuming that one egg was laid per day (e.g., Kingma et al. 2009). The onset of incubation was determined by daily nest-box visits from the date the seventh egg was laid onwards. The onset of incubation was defined as the first day the female was found incubating or the first day the eggs were found uncovered and warm. A few days before the expected hatch date (~day 11 of incubation onset), nest-boxes were visited daily to determine hatch date (hatching day 1 is the day of the hatching of the first egg).

We attempted to catch the male and female breeding in each nest-box when they were feeding their nestlings (mostly 7–8 days old) using a ‘flap-trap’ inside the nest-box. Each adult was marked with an individually numbered metal ring. Individuals were aged in the field using plumage characters (classifying them into 1 year old individuals (yearlings) or adults) according to Svensson (1992), and reliably sexed according to the presence or absence of a brood patch (only females develop a brood patch). The age of ringed adults was estimated based on ringing records since 2001. In a small number of cases, individuals (mostly unringed birds, presumably immigrants) were first caught with adult plumage (12.7 % of 102 females and 8.0 % of 87 males in our dataset). In these cases, we assigned them to 2 years old as older individuals were relatively rare (see below). Due to the low number of old individuals (>2 years) in different age classes, the age classes were divided into three age groups: 1 year old (56.9 % females and 57.5 % males), 2 years old (29.4 % females and 27.6 % males) and ≥3 years old (13.7 % females and 14.9 % males).

### Measurements of female incubation behaviour

Female incubation behaviour was recorded from day 5 of the incubation onset onwards in 92 nest-boxes. On day 4, temperature loggers (Thermochron iButtons, Maxim integrated products) were placed in each nest between the eggs. These temperature loggers were set to record temperature at 3-min intervals. With this interval, it was possible to monitor the female incubation behaviour for circa 4 consecutive days. Other studies found no effect of temperature loggers on female incubation behaviour in songbirds (e.g. Weidinger 2008). We did not observe iButton removal from the nest-box but in only three nest-boxes, the blue tits buried the logger deep inside the nest-material and we excluded these nests from our data analyses.

By using the fluctuations in temperatures recorded by the temperature loggers, it is possible to determine when a female left or returned to the nest. The rhythm programme (1.1; Cooper and Mills 2005) was used to select incubation recesses from the temperature records, as defined when the temperature inside the nest dropped by more than 2 **°**C and the declining trend in temperature lasted more than 2 min. The Raven Pro programme was used (1.5; Cooper and Mills 2005) to visually inspect the time series. Furthermore, the recesses in the time series were manually selected if they had not been selected by rhythm (e.g. a sharp drop in temperature that lasted less than 2 min). From these data, it is possible to determine the time of onset and calculate the duration of each recess and female attentiveness bout. To validate the data on nest attentiveness derived from the temperature loggers, 10 nest-boxes were randomly selected and female nest attentiveness recorded by infra-red cameras in the nest-boxes calculated (mean total observation duration = 7.68 h). The female nest attentiveness data obtained by using the camera (using ‘BirdBox’; see below for detailed procedures) were highly correlated with the data obtained by using temperature logger data (*r* = 0.74, *p* = 0.014).

Because during the night females always incubate inside the nest-box, incubation behaviour patterns for this study were calculated from sunrise until sunset. For analyses, the total number of minutes the female was on the nest (on-bouts, female nest attentiveness), the mean length of recesses (off-bouts, mean time females spent off-nest per bout) and minimum daily nest temperature were calculated. To minimize observer bias, blinded methods were used for the analysis of female incubation behaviour.

### Measurements of male incubation feeding

The male incubation feeding behaviour inside the 63 nest-boxes was recorded with infra-red cameras placed directly under the lid of the nest-box. We did not have enough cameras to record male incubation feeding inside all 92 nest-boxes. In order to let the birds grow accustomed to the presence of the camera, dummy cameras were placed in the nest-boxes before egg laying. On day 5 after incubation onset, the dummy cameras were replaced by real cameras and on day 6 in the morning, recording was started and continued for circa ~ 8 h.

The video data were analysed with the programme BirdBox (HH, unpublished). The programme calculates the picture moments *M*_*ij*_, *i*,*j* = {0,1,2,3}, and the luminance-histogram for each frame, the running average of frames and the total average of all frames in a video. The programme then calculates the mean square errors of the moments of each frame with the moments of the previous frame, the running average and the total average. Similarly, the *Χ*^2^ distances of the histograms are calculated. From these signals, BirdBox generates a graph with time on the *x*-axis and ‘activity’ on the *y*-axis, with peaks most likely at entry/exit events. This time-line is displayed together with the actual video footage to guide the user finding relevant sequences in the footage. The user annotates events and sequences directly in the time-line. From this, BirdBox generates a summary of the outputs including male incubation feeding (counts per hour), female nest attentiveness (minutes per hour) and female recess length (minutes). BirdBox uses the hardware accelerated H.264 video decoder of NVidia GPUs and CUDA for image analysis.

The duration the female is on the nest may affect the opportunity for the male to feed the female on the nest. Therefore, incubation feeding (number of times the male feed the female) was defined as the number of feeds while the female was inside the nest-box. There was not any male entry into the nest-box when females were not present in the nest-box. To minimize observer bias, blinded methods were used for the analysis of male incubation feeding.

In addition to infra-red cameras inside the nest-boxes, we also recorded the behaviour with conventional cameras in front of 10 nest-boxes (5–10 m distance, ~2 h per nest-box), to record if males fed females on and surrounding the nest-box. We did, however, not record any feedings by males to females outside the nest-box this way. For 15 nest-boxes, we also followed the female further away with binoculars but we could not determine the number of male feedings that may have occurred away due to difficulties to follow these birds in the dense vegetation (see Nilsson and Smith 1988; Pearse et al. 2004; Matysioková and Remeš 2010).

### Measurements of ambient temperature

Ambient temperatures were obtained during the recording hours of incubation behaviour and incubation feeding from the meteorological station located in Eelde Airport, Groningen, close (1.6 km) to the study area. The average daily temperature was calculated from sunrise until sunset for the days of incubation behaviour recording (circa 4 consecutive days) per nest box. For the incubation feeding behaviour, the average daily temperature per nest-box was used from the beginning until the end of recording (circa 8 h).

### Measurements of habitat quality

Habitat quality was measured following an established protocol for the study area (for details see Amininasab et al. 2016) by measuring the tree sizes surrounding the nest-boxes (*n* = 92). For evaluating the radius around each nest-box in which vegetation structure may be relevant, the radius of 20 m was chosen as a compromise between biological meaning (the distance blue tits can forage from the nest-box) and practical feasibility (the workload of counting and measuring trees) (Amininasab et al. 2016). Within the 20 m radius of nest-boxes, we sampled and identified the species of all living trees with a minimal circumference of 30 cm. Trees with thinner trunks were excluded as they were not considered to harbour a substantial amount of food for breeding blue tits. The trees’ circumference (cm) was measured about 130 cm from the ground, using a measuring tape. We identified and measured 3727 trees surrounding the selected nest-boxes. We used the counts of trees including deciduous (*n* = 3209) and coniferous species (*n* = 518) surrounding the nest-boxes as habitat variables which represent food availability around the nest-sites. We also calculated the proportion of deciduous trees, defined as the number of all deciduous trees divided by the total number of all trees, in the 20 m radius around the nest-box as an index of habitat quality. The most dominant species surrounding the selected nest-boxes was English oak *Quercus robur* (*n* = 1047), a deciduous species and an important source of the main prey item for blue tits, caterpillars, during the breeding season (Blondel et al. 1991; Perrins 1991). Hence, the density of English oak trees was also defined as a separated index of habitat quality.

### Ethical considerations

This study conforms to the animal welfare standards, and all experiments approved by the animal experimentation standards of the University of Groningen (DEC-6367). Data obtained from the temperature data loggers and red-vision cameras indicated that in most cases the birds returned to the nest. We used standard methods in capturing and handling adults in the nest-box. The duration of placing the temperature loggers, setting cameras and handling adults was kept to a minimum to minimize stress for the individuals.

### Statistical analyses

For the statistical analyses, we used the programme R (version 3.1.0.; R Development Core Team 2014). Two types of models were applied to analyse the relationships between environmental and individual parameters (ambient temperature, habitat quality and age of each individual) and patterns of female incubation behaviour (female nest attentiveness, length of recess and minimum nest temperature) or male incubation feeding frequency. Model residuals were always checked to confirm assumptions of normality. First, we applied linear models with normally distributed errors to test the relationships between patterns of female incubation behaviour (response variable) and environmental parameters and age of the female. The length of recesses was log transformed to ensure normality of the data. Second, for analyses of male incubation feeding, a generalized linear model with Poisson distribution (offset = total female incubating time) was used with male incubation feeding as response variable, and ambient temperature, habitat quality, age of male and age of female partner as predictors. In addition, Pearson correlations were used to explore singular correlations between variables and to detect collinearity between variables. As the density of English oak trees was positively correlated with proportion of deciduous trees surrounding the nest-boxes (*r* = 0.33, *n* = 92, *p* = 0.001), the proportion of deciduous trees was included as an index of habitat quality in the models. Non-significant terms (*p* > 0.05) were removed using stepwise deletion until the minimal model was produced. Statistical parameters were presented based on the minimal model with only significant predictors, and parameters of non-significant predictors were obtained immediately before they were removed from the model.

## Results

### Female incubation behaviour

Female blue tits left the nest-boxes with off-bouts averaging 8.91 min (SD = 1.80, *n* = 92). The mean minimum nest temperature was 33.29 **°**C (SD = 1.80). The total mean female nest attentiveness was 42.15 (SD = 4.38) min per hour. The average length of recesses per female was negatively correlated with the minimum nest temperature (*r* = −0.32, *n* = 92, *p* = 0.002) and total nest attentiveness (*r* = −0.23, *n* = 92, *p* = 0.026).

**Fig. 1:**
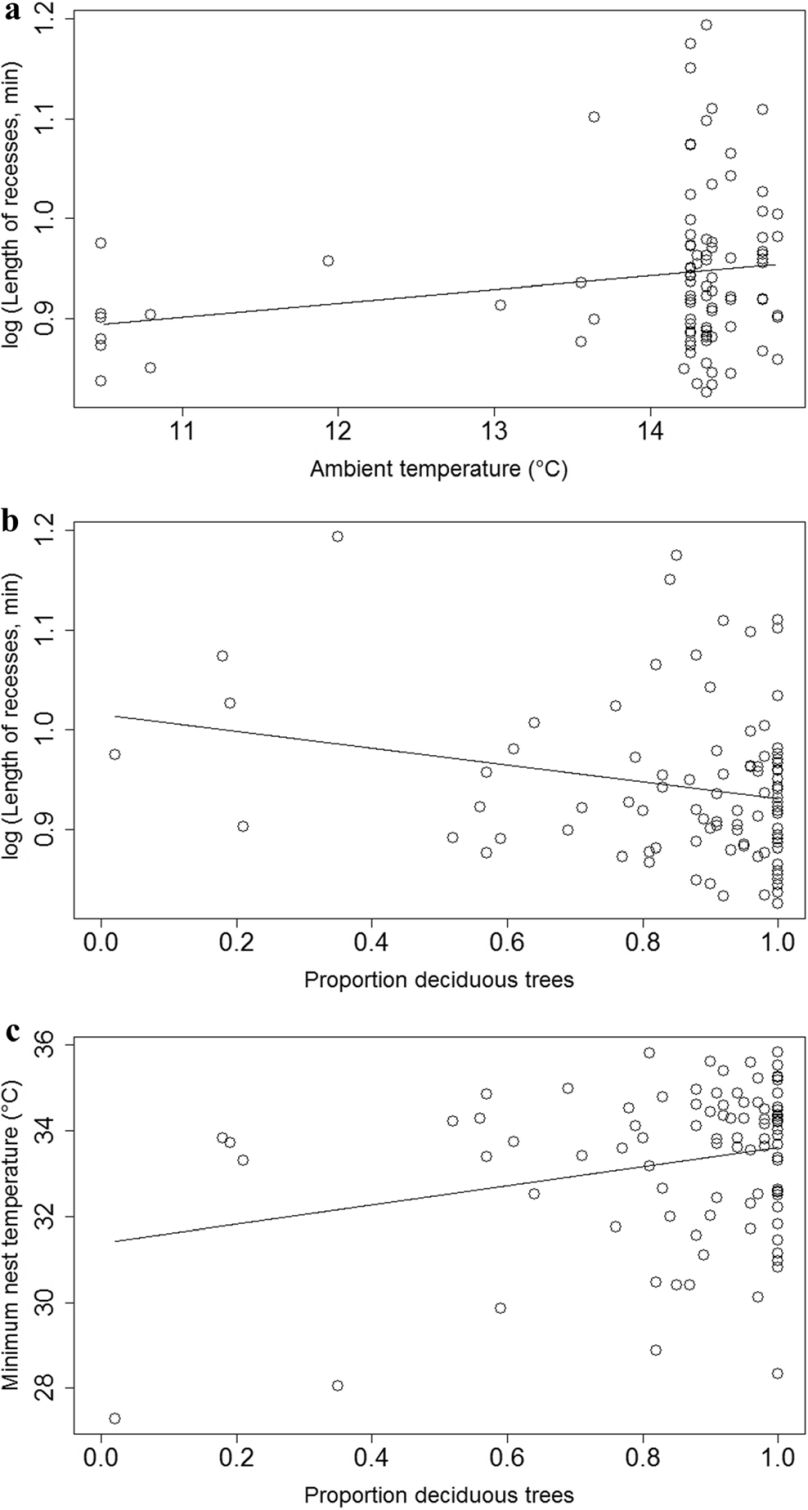
The effect of ambient temperature and proportion of deciduous trees on patterns of female incubation behaviour. **a, b** The relationship between the length of female recess (min) with ambient temperature (**°**C, Estimate ± SE = 0.014 ± 0.007, *t* = 1.94, *n* = 92, *p* = 0.055) and proportion of deciduous trees (Estimate ± SE = −0.084 ± 0.040, *t* = −2.11, *n* = 92, *p* = 0.037) surrounding the nest-boxes, respectively. **c** The relationship between the minimum nest temperature (**°**C) and proportion of deciduous trees (Estimate ± SE = 2.226 ± 0.909, *t* = 2.45, *n* = 92, *p* = 0.016) surrounding the nest-boxes

**Fig. 2:**
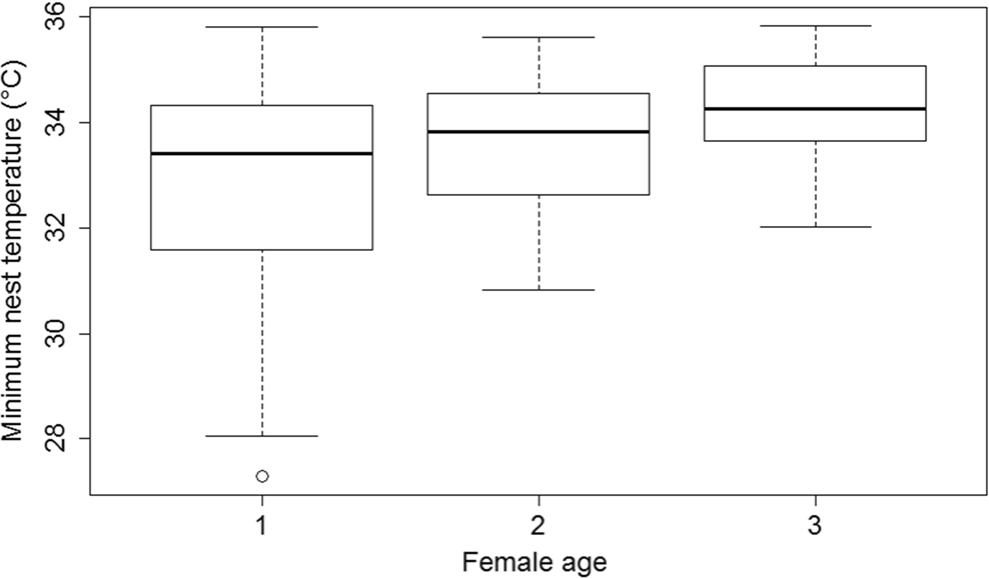
The plotted minimum nest temperature (median, percentiles and quartiles) in female blue tits of different age groups (*n* = 52, 25, 11 for 1, 2 and 3 years old, respectively) over the incubation period. *Circles* represent data more than 1.5 IQR below and above the first and third quartile

Females took significantly shorter recesses when the ambient temperature was lower and also when the proportion of deciduous trees surrounding the nest-boxes was higher (Table 1, Fig. 1a, b). The ambient temperature was not correlated with proportion of deciduous trees (*r* = 0.09, *n* = 88, *p* = 0.50). There was no significant effect of female age on the length of recesses (Table 1). None of the variables investigated were associated with total female nest attentiveness (Table 1). The minimum nest temperature was higher when the ambient temperature and the proportion of deciduous trees surrounding the nest-boxes were higher (Table 1, Fig. 1c) and when females were older (Table 1, Fig. 2).

**Table 1:** Linear model analyses of factors predicting length of recesses (min), female nest attentiveness (min/h) and minimum nest temperature (**°**C) over the incubation period (*n* = 88^a^) in blue tits

	Length of recesses (min)			Female nest attentiveness (min/h)			Minimum nest temperature (**°**C)		
Predictors	Estimate ± SE	*t* value	*p* value	Estimate ± SE	*t* value	*p* value	Estimate ± SE	*t* value	*p* value
(Intercept)	0.813 ± 0.109	7.429	<0.001	49.387 ± 6.368	7.756	<0.001	25.072 ± 2.377	10.546	<0.001
Ambient temperature (**°**C)	*0.015 ± 0.007*	*1.990*	*0.049*	−0.546 ± 0.447	−1.221	0.23	*0.399 ± 0.167*	*2.388*	*0.019*
Proportion deciduous trees	*−0.095 ± 0.040*	*−2.335*	*0.021*	1.680 ± 2.367	0.710	0.48	*1.948 ± 0.884*	*2.204*	*0.030*
Female age	0.000 ± 0.012	0.034	0.97	−0.629 ± 0.690	−0.910	0.36	*0.585 ± 0.258*	*2.269*	*0.025*

Estimated effect sizes of each term (Estimate) with associated standard errors, *t* and *p* values were presented based on the minimal adequate model in italics, and parameters of non-significant predictors were obtained immediately before they were removed from the model

^a^Among 92 nest-boxes with temperature logger data, the age of females was unknown in four nest-boxes and these four were therefore not included

### Male incubation feeding behaviour

**Fig. 3:**
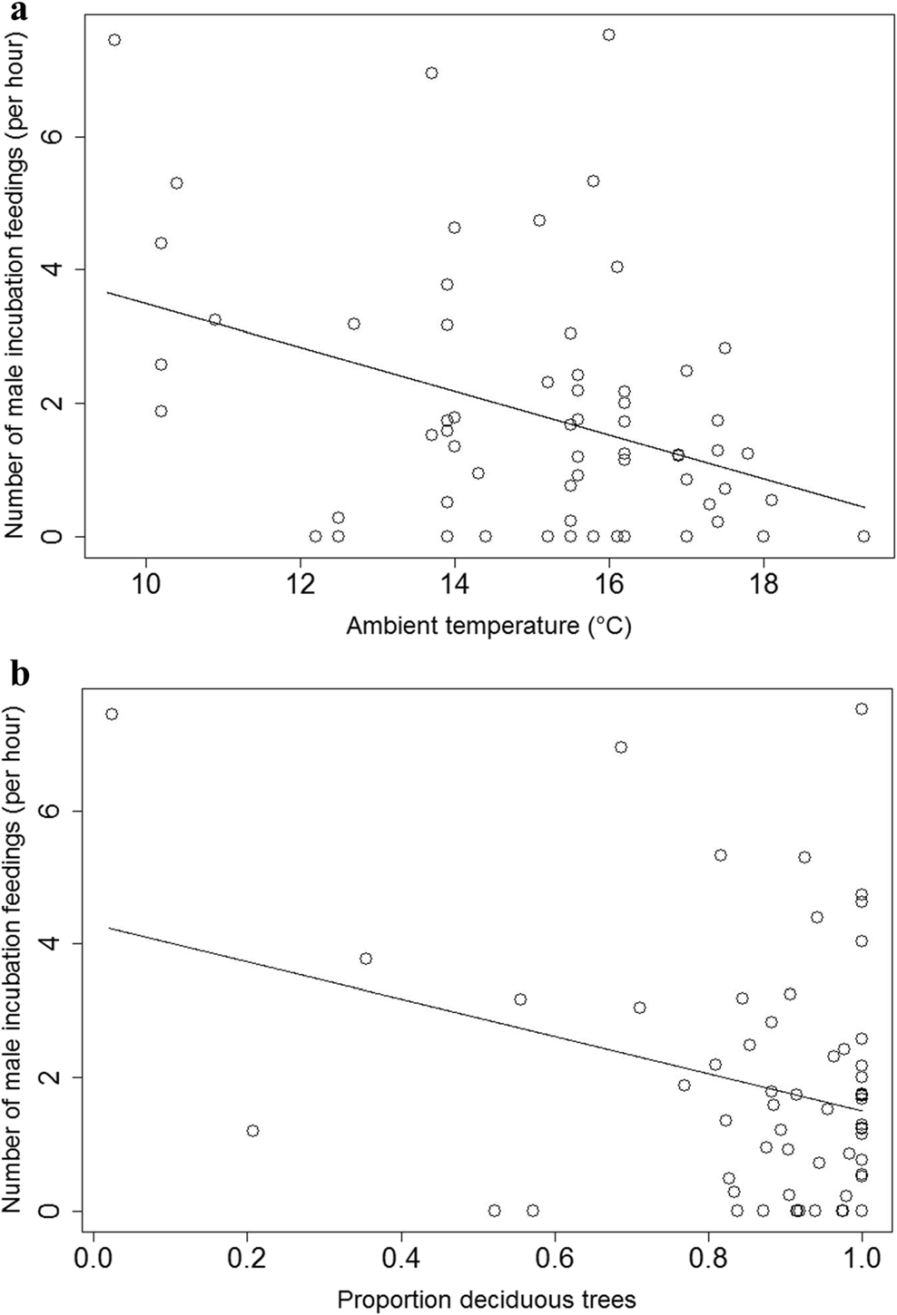
The relationships between the frequency of male incubation feedings per female attentive hour and the **a** ambient temperature (Estimate ± SE = −0.328 ± 0.102, *t* = −3.20, *n* = 63, *p* = 0.002) **b** proportion of deciduous trees (Estimate ± SE = −2.793 ± 1.200, *t* = −2.33, *n* = 63, *p* = 0.023) surrounding the nest-boxes over the incubation period

Males fed the females on average 1.84 times per hour (SD = 1.89, range = 0–7.52, *n* = 63). The frequency of male incubation feeding during the time the female was in the nest-box decreased with higher ambient temperatures and with a higher proportion of deciduous trees surrounding the nest-boxes (Table 2, Fig. 3a, b). There was no effect of male age and age of his female on intensity of male incubation feeding surrounding the nest-boxes with a higher proportion of deciduous trees (Table 2).

**Table 2:** General linear model analysis with a Poisson distribution of factors predicting the intensity of male incubation feeding per female attentive hour (*n* = 53 ^a^)

	Number of male incubation feedings per female attentive hour		
Predictors	Estimate ± SE	*z* value	*p* value
(Intercept)	2.855 ± 0.278	10.262	<0.001
Ambient temperature (**°**C)	*−0.120 ± 0.019*	*−6.323*	*<0.001*
Proportion deciduous trees	*−0.550 ± 0.186*	*−2.956*	*0.003*
Male age	−0.038 ± 0.082	−0.464	0.64
Partner (female) age	−0.071 ± 0.069	−1.026	0.30

Estimated effect sizes of each term (Estimate) with associated standard errors, *z* and *p*-values were presented based on the minimal adequate model in italics, and parameters of non-significant predictors were obtained immediately before they were removed from the model

^a^Among 63 nest-boxes for which we recorded male incubation feeding, age of one or both parents were unknown for ten nest-boxes and these were therefore not included

**Fig. 4:**
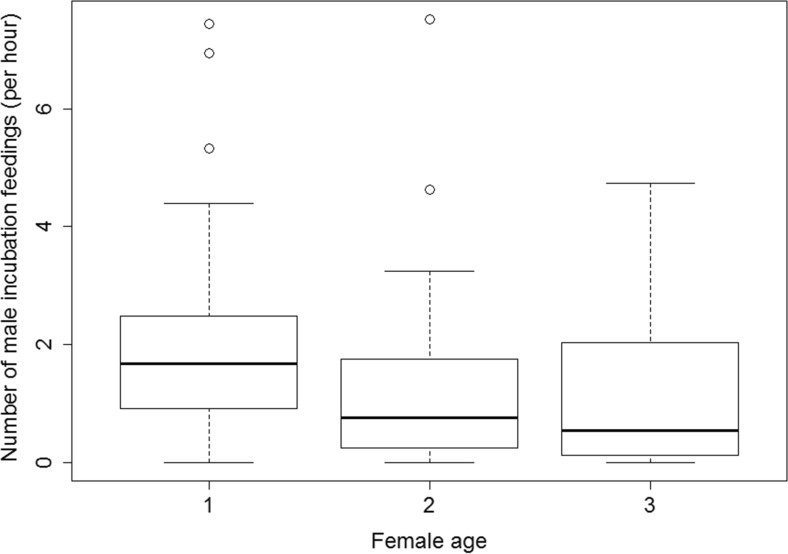
The plotted intensity of male incubation feedings per female attentive hour (median, percentiles and quartiles) in females of different age groups (*n* = 37, 15, 7 for 1, 2, and 3 years old, respectively) over the incubation period. *Circles* represent data more than 1.5 IQR below and above the first and third quartile

As female age was collinear with proportion of deciduous trees (*r* = 0.29, *n* = 53, *p* = 0.037), we tested an alternative model only investigating the effect of female age on male incubation feeding which suggests that males increased the intensity of incubation feeding when their partners were younger (Fig. 4, Estimate ± SE = −0.178 ± 0.066, *z* = −2.711, *n* = 59, *p* = 0.006). Male age did not significantly correlate with the proportion of deciduous trees (*r* = 0.18, *n* = 53, *p* = 0.19).

## Discussion

### Female incubation behaviour

Our results show that ambient temperature is an important predictor of female incubation behaviour. Although total female nest attentiveness was not associated with ambient temperature, females modified the length of each recess bout depending on the temperature. Lower temperatures caused females to spend less time off the nest per bout. This effect is similar as in other species (Biebach 1986; Conway and Martin, 2000; Boulton et al. 2010) and is most likely the consequence of potential harmful effects on the developing embryos if temperature drops below a certain level (Boulton et al. 2010). More frequent re-heating of eggs and shorter off-nest bouts likely buffer the faster drop of egg temperature at lower ambient temperatures (as shown in great tits: Boulton and Cassey 2012). In addition, prolonged recess would result in a net loss of female energy for re-warming the (colder) eggs (Camfield and Martin 2009). However, there are studies which found no effect of ambient temperature on egg temperature (e.g. Bogdanova et al. 2007). Hence, these patterns can vary depending on species, populations and environment.

Habitat quality, another important parameter of environmental characteristics, may influence incubation behaviour because this may alter the trade-off between self-maintenance and investment in the brood (Henson and Cooper 1993; Zicus et al. 1995; Rauter and Reyer 1997; MacCluskie and Sedinger 1999; Manlove and Hepp 2000). For example, female nest attentiveness can increase in better quality habitats, because females using high-quality habitats are better or faster able to satisfy food requirements (Rauter and Reyer 1997; Zanette et al. 2000; Eikenaar et al. 2003; Zimmerling and Ankney 2005; Duncan-Rastogi et al. 2006; Chalfoun and Martin 2007). Therefore, higher food availability enables females to spend more time keeping eggs at optimal temperatures which may reduce the costs of incubation (Londono et al. 2008). However, in our population, similar to Matysioková and Remeš’s (2010) study on great tits, female nest attentiveness was not directly related to habitat quality. However, females took shorter off-bout length in high-quality habitats, suggesting that females presumably were indeed able to find their food resources faster in these higher quality habitats.

In addition to environmental conditions, female age can influence incubation behaviour because older females may be more efficient foragers and/or incubators. In our study population, there was no effect of female age on the length of recesses or female nest attentiveness. However, female age was positively associated with nest temperature. This result indicates that younger females are less capable of keeping eggs warm, which may be due physiologically characteristics and/or energy constraints (Aldrich 1983; Yerkes 1998). Similar to our study, female age is not related to female nest attentiveness in glaucous-winged gulls *Larus glaucescens* (Reid 1988). In contrast to our study, in herring gulls *Larus argentatus* female age does not correlate with egg temperature (Bogdanova et al. 2007). This illustrates that, overall, there are not enough studies to draw conclusions about age-specific patterns of incubation behaviour across species, and more longitudinal studies are needed to test the generality of our results to unveil the mechanisms underlying the link between female age and patterns of incubation behaviour in birds.

### Male incubation feeding

Our results show that the frequency of male incubation feeding during the time the female was in the nest-box decreased with higher ambient temperatures. Furthermore, the intensity of male incubation feeding was lower in nest-boxes around which the proportion of deciduous trees was higher. This shows that males lower their intensity of incubation feeding when environmental temperature and food conditions are favourable and under which females can reduce the duration of incubation bouts (Lifjeld and Slagsvold 1986; Lifjeld et al. 1987; Smith et al. 1989; Kovařík et al. 2009). However, several studies showed opposite results. For example, Zanette et al. (2000) found that male Eastern yellow robins *Eopsaltria australis* fed their incubating females more in high-quality habitats. In a study on great tits, Matysioková and Remeš (2010) indicated that male incubation feeding increased with habitat quality, but only in years with low food availability. The lower intensity of male incubation feeding in high-quality habitats in our study indicates that likely, the area offered sufficient food resources to enable females to fulfil food requirements themselves. However, in agreement with Matysioková and Remeš’s (2010) study on great tits, our findings are based on male incubation feeding inside the nest-box. Although we did not record any incubation feedings in close proximity of the nest-box, we were not able to measure the incubation feeding rate further from the nest-box, which, if this happens, may obscure habitat quality effect on overall incubation feeding.

Furthermore, food availability usually covaries with ambient temperature (Londono et al., 2008), and because of this, we cannot separate the effect of ambient temperature from habitat quality. Regardless of the mechanism, our results suggest that males feed the incubating females more when females need to incubate more (lower ambient temperature) and/or cannot obtain enough food themselves during off-bouts (i.e. in low-quality habitats with more coniferous habitat and when ambient temperature is lower).

The frequency of incubation feeding in our study population was independent of male age, which was also found in great tits (Matysioková and Remeš 2010). This was in contrast to our expectations, because we expected an increase in incubation feeding with male age due to improved experience and foraging efficiency. In spite of this, males only increased the frequency of incubation feeding when their partners were younger or where the female partners occupied the low-quality habitats with less food availability. This indicates that males likely monitor their female’s needs to fulfil sufficient parental care (Houston et al. 2005). Moreover, investigating males’ incubation feeding in response to certain characteristics of both males and females can help to determine how they can adjust the parental care based on the energy constraints, and this can in turn have consequences for life history evolution and sexual selection (Deeming 2002; Martin 2002). However, there are very few studies which indicate how incubation feeding varies with male age and the age and quality of female partner. Hence, more studies are needed to clarify the general effect of male and partner age on incubation feeding within and between species of birds.

### Implications for avian life history strategies

Birds providing parental care need to optimize their investment according a trade-off between reproductive output and self-maintenance, and this becomes especially prominent in those species that incubate during periods with different temperatures or in habitats with different quality (Ardia and Clotfelter 2007). We show here that, for both males and females, this trade-off depends on intrinsic (e.g. age or quality) and environmental characteristics (e.g. ambient temperature and habitat quality), which determine how individuals can deal with the energetic constraints and brood demand during incubation. These results help to increase our understanding of the energetic costs of incubation and explain how important life history strategies can vary based on a suite of characteristics that determine both male and female strategies, and the interplay between those, to optimize reproductive performance. Our study also indicates that plasticity in female incubation behaviour and male incubation feeding can explain how birds cope with the climatic and foraging conditions to improve their fitness and adjust the trade-off between own energetic needs (e.g. foraging and self-maintenance) and the thermal requirements of the developing embryos. However, future experimental work on this species manipulating different intrinsic and environmental conditions may reveal further insight into the incubation strategies and the energy constraints hypothesis.

## Conclusion

We showed that an increase in ambient temperature resulted in a higher nest temperature, and this enabled incubating blue tit females to increase self-maintenance activities off the nest. Probably as a consequence of this, an increase in ambient temperature was associated with fewer incubation feedings by the male. Similarly, in areas with more deciduous trees, females had shorter duration of recesses and males fed incubating females less. Males also increased the frequency of incubation feeding when their partners were young, probably to improve incubation efficiency of such females who had nests with lower temperatures. In conclusion, the patterns of incubation behaviour and incubation feeding were related to both environmental and individual characteristics, which can facilitate new insights to the study of avian incubation energetics and how species differ in patterns of parental care.
